# Chromosome-Y haplogroups in Asturias (Northern Spain) and their association with severe COVID-19

**DOI:** 10.1007/s00438-024-02143-4

**Published:** 2024-05-04

**Authors:** Mar González-Fernández, Daniel Vázquez-Coto, Guillermo M. Albaiceta, Laura Amado-Rodríguez, Marta G. Clemente, Lucinda Velázquez-Cuervo, Claudia García-Lago, Juan Gómez, Eliecer Coto

**Affiliations:** 1 Genética Molecular, Hospital Universitario Central Asturias, Oviedo, Spain; 2 Instituto de Investigación Sanitaria del Principado de Asturias, ISPA, Oviedo, Spain; 3 Unidad de Cuidados Intensivos Cardiológicos, Hospital Universitario Central Asturias, Oviedo, Spain; 4 Universidad de Oviedo, Oviedo, Spain; 5 CIBER-Enfermedades Respiratorias, Instituto de Salud Carlos III, Madrid, Spain; 6 Instituto Universitario de Oncología del Principado de Asturias, Oviedo, Spain; 7 Neumología, Hospital Universitario Central Asturias, Oviedo, Spain

**Keywords:** Chromosome-Y, Haplogroups, COVID-19, Hypertension

## Abstract

The main objective of this study was to determine whether the common Y-haplogroups were be associated with the risk of developing severe COVID-19 in Spanish male. We studied 479 patients who required hospitalization due to COVID-19 and 285 population controls from the region of Asturias (northern Spain), They were genotyped for several polymorphisms that define the common European Y-haplogroups. We compared the frequencies between patients and controls aged ≤ 65 and >65 years. There were no different haplogroup frequencies between the two age groups of controls. Haplogroup R1b was less common in patients aged ≤65 years. Haplogroup I was more common in the two patient´s groups compared to controls (*p* = 0.02). Haplogroup R1b was significantly more frequent among hypertensive patients, without difference between the hypertensive and normotensive controls. This suggested that R1b could increase the risk for severe COVID-19 among male with pre-existing hypertension. In conclusion, we described the Y-haplogroup structure among Asturians. We found an increased risk of severe COVID-19 among haplogroup I carriers, and a significantly higher frequency of R1b among hypertensive patients. These results indicate that Y-chromosome variants could serve as markers to define the risk of developing a severe form of COVID-19.

**Supplementary Information:**

The online version contains supplementary material available at 10.1007/s00438-024-02143-4.

## Introduction

The main objective of this study was to determine whether the common European chromosome Y-haplogroups were associated with the risk of developing a severe form of COVID-19.

The coronavirus disease-2019 (COVID-19) caused by the SARS-Cov-2 is characterised by a wide array of symptoms: while many individuals remain asymptomatic or showed a mild disease, others develop a severe pneumonia and complications in other organs that require hospitalization and increase the risk of death (Zeng et al. 2021). Risk factors such as advanced age, male sex, obesity, pre-existing cardiovascular and lung disease, have been associated with a higher risk of developing severe COVID-19 (Holt et al. 2022; Coto et al. 2021).The heterogeneous symptoms could be partially explained by the individual’s genetic background, and several genetic variants have been associated with the risk of developing severe COVID-19 and the risk of death (Ellinghaus et al. 2020; Cuesta-Llavona et al. 2021; Butler-Laporte et al. 2022). Male are at increased risk of developing severe COVID-19 compared to female and sex-specific genetic factors could thus contribute to the extent of disease symptoms (Peckham et al. 2020; Cruz et al. 2022).

Genes that regulate the immune response against viral infection are strong candidates to be associated with the risk of severe COVID-19 (Asano et al. 2021). Studies in rodents support that Y-chromosome genes play a role in the variability found between male and female immunity (Teuschert et al. 2006; Wesley et al. 2007). Animal studies also showed that Y-chromosome plays a significant role in the regulation of blood pressure and T-cell infiltration in tissues key to the cardiovascular function (Davidson et al. 1995; Khan et al. 2019). Y-chromosome variation in a murine model influence pathogenesis of the influenza A virus, increasing the susceptibility to the virus, immune response in lungs and T-lymphocyte activation, with higher IL-17 levels (Krementsov et al. 2017). In this regard, the presence of chromosome Y could explain in part the higher risk of severe COVID-19 among male. Y-chromosome could also contribute to COVID-19 through the risk of developing cardiovascular traits that increase the risk of suffering a severe form of the disease (Kloc et al. 2020).

Blood cells from male who developed a severe disease showed higher frequency of loss of Y (LOY) compared to healthy donors, and LOY has been associated with changes in immune gene transcription and increased risk for mortality in several diseases (Barros et al. 2020; Loftfield et al. 2019). LOY may also be associated to increased genome instability and impaired leucocyte immune responses (Thompson et al. 2019; Dumanski et al. 2021). At least one study reported loss of Y in leukocytes as a risk factor for critical COVID-19 in men (Bruhn-Olszewska et al. 2022).

Approximately 95% of the Y-sequence does not undergo recombination with the X-chromosome and is thus transmitted in full from father to son. These non-recombinant regions have accumulated nucleotide changes that originated in a particular population and passed down from a common male ancestor, spreading around the world through migrations. Groups of different Y-single nucleotide polymorphisms (SNPs) define the Y-haplogroups, with frequencies that are characteristic of each human population. For example, Y-R (defined by SNP M207 = rs2032658) originated about 30 thousand years ago and is the prevalent haplogroup among Europeans. More than half of the Spanish male carry haplogroup Y-R1b, defined by SNPs rs2032624 (R1) + rs9786153 (R1b), which had its origin in the upper paleolithic (approx. 25,000 years ago) in the Caucasus region (Myres et al. 2011). A sub-lineage of R1b known as DF27 (R1b1a2a1a2a) surged about 4200 years ago and is currently the prevalent among Spanish male (Solé-Morata et al. 2017; Villaescusa et al. 2017). Therefore, the analysis of this Y-variants allows to obtain information about population events throughout human history (Jobling and Tyler-Smith 2003; Parker et al. 2020; Underhill et al. 2000).

Y-haplogroups have been also associated with differences in inflammatory responses and cytokine production and several studies found significant association between some haplogroups and cardiovascular traits (Khan et al. 2019; Bloomer et al. 2014; Maan et al. 2017; Kuroki and Fukami 2023). Haplogroup I has been associated with coronary artery disease (CAD), and men with this haplogroup presented changes in expression of genes involved in atherosclerosis, response to viral infection, lipid metabolism, coagulation and haemostasis (Charchar et al. 2012; Bloomer et al. 2013; Voskarides et al. 2014; Eales et al. 2019; Maan et al. 2017; Lorca et al. 2023). Y-I was also associated with a lower immune response and a faster disease progression and mortality in HIV infected men (Sezgin et al. 2009).

Based on the role of Y-chromosome in the extent of immune responses and the risk of cardiovascular traits, we hypothesised that Y-haplogroups could explain in part the risk of developing severe COVID-19 among male. Thus, we evaluated the association between the common European Y-haplogroups and severe COVID-19 in a cohort of Spanish male.

## Material and methods

### Study subjects

The study was approved by the Ethics Committee of Principado de Asturias (Oviedo, Spain) and all the participants gave their consent to participate in the study. All the participants were > 18 years old and of Spanish ascent residents in the region of Asturias (northern Spain, total population 1 million).

We studied 479 male patients who required hospitalization due to COVID-19 between March 2020 and April 2021. They were followed till disease remission with hospital discharge or death. Data about pre-existing hypertension was obtained from the clinical history at hospital admission. Following previously reported criteria we considered early-onset COVID-19 as an age at hospitalization ≤65 years (Niemi et al. 2022).

The controls (*N* = 285; 38% ≤65 years) were recruited from the general population with the main purpose of defining the Y-variant frequencies in our region. Although the existence of SARS-CoV-2 infection was not determined in the controls, none of them required hospitalization due to COVID-19. The main values in the study cohort are summarised in Table 1.

**Table 1:** Main values in the COVID-19 patients

Total	479
Mean age (years)	63.64
Range (years)	21–93
≤65 years	248 (52%)
>65 years	231
Dyslipemia yes	204 (43%)
Dyslipemia no	275
Diabetes yes	90 (19%)
Diabetes no	389
Hypertension yes	247 (52%)
Hypertension no	232
Death	56 (12%)
Survivors	423

### Y-haplogroups determination

All the participants were genotyped for single nucleotide polymorphisms (SNPs) that defined the most common European Y-haplogroups (Table 2). The nucleotides were determined by real-time PCR with Taqman assays in an ABI7500 equipment and following the manufacturer instructions (Fisher Scientific). The allele frequencies among Europeans were obtained from the Ensembl database (www.ensembl.org).

**Table 2:** The 9 single nucleotide polymorphisms determined to define the Y-haplogroups, genotyped with Taqman assays (Fisher scientific) or Sanger sequencing of PCR fragments (R1b-DF27)

SNP ID	Haplogroup	Taqman assay ID	Europeans allele frequency
rs2032658 G/A	R	C_2307221_1Y	G (0.58)
rs2032624 C/A	R1	C_2292796_20	C (0.58)
rs9786153 T/C	R1b	C_29812961_10	C (0.53)
rs2534636 C/T	R1a	C_26236081_10	T (0.05)
rs577478344 A/G	R1b-DF27	Sanger sequencing	A (0.08)
rs2032597A/C rs111665403 A/G	I	C_1083231_10 Sanger sequencing	C (0.14) G (0.14)
rs13447352 A/C	J	C_33589462_10	C (0.11)
rs9306841 C/G	E	C_29796914_10	G (0.03)
rs2032636 G/T	G	C_2292797_20	T (0.06)

The frequency of each allele that defines the haplogroups among Europeans was obtained from the Ensembl portal (www.ensembl.org)

The genotyping of the DF27 sub-clade (SNP rs577478344 A/G) was performed by PCR with primers specific for the target region 5′TGTTAAAGTCCTGCGCTATTATGGTGT and 5′AAATATAGACGAATGCATAACTAGAATAACC, followed by Sanger sequencing in a capillary electrophoresis equipment (ABI3130xl, Thermo Fisher) (Suppl. Figure 1).

The presence of haplogroup I was confirmed by Sanger sequencing of PCR fragments containing the rs111665403 SNP, in complete linkage disequilibrium with rs2032597 (Suppl. Figure 2).

### Statistical analysis

Statistical analysis was performed with R (version R-4.3.3 for Windows; https://www.r-project.org). All the values were collected in an excel file. Descriptive data are presented as total values and %. Patients and controls were classified as ≤65 and >65 years and the Y-haplogroup frequencies were compared between the groups with logistic regression. A *p* value below 0.05 was considered significant. Odds ratio´s (OR) and 95% confidence intervals (CI) were also calculated.

The presence of hypertension and the association of this variable with the Y-haplogroups was also determined in patients and controls.

## Results

The prevalence of the different Y-chromosome haplogroups in the Asturias controls and COVID-19 patient’s are shown in Table 3. As expected, haplogroup R was the most frequent in our population with haplogroup R1b present in 66% and 63% of the controls >65 and ≤65 years, respectively (*p* = 0.25). There were no difference for the other haplogroups between the two control age-groups. The Y-R1b was significantly decreased in the younger vs. older patients (59% vs. 69%; *p* = 0.007; OR = 0.60, 95%CI = 0.41–0.87). We compared the patients with their age-matched controls. Y-R1b frequencies were no different between patients and controls ≤60 years (*p* = 0.78), and >60 (*p* = 0.52) (Table 3). We determined the frequency of the DF-27 subtype (SNP rs577478344) by sequencing all the R1b patients and controls. DF-27 was present in 83% and 84% of the R1b controls aged ≤60 and >60 years, and in 81% and 82% of the R1b patients aged ≤60 and >60 years. Thus, there were no difference for the DF-27 frequencies between the R1b groups but this marker was significantly less frequent in total patients ≤65 vs. >65 years (Table 3).

**Table 3:** Haplogroup frequencies for the two age groups of hospitalized COVID-19 patients and controls

	≤65 years		>65 years	
	Patients (*N* = 248)	Controls *N* = 107	Patients (*N* = 231)	Controls *N* = 178
Haplogroup				
R	148 (59%)	68 (64%)	164 (71%)	120 (67%)
E	25 (10%)	10 (9%)	13 (6%)	18 (10%)
G	5 (2%)	7 (7%)	7 (3%)	11 (6%)
I	23 (9%)	5 (5%)	19 (8%)	7 (4%)
J	18 (7%)	10 (9%)	9 (4%)	12 (7%)
Other	29 (12%)	7 (7%)	19 (8%)	10 (6%)
R-subhaplogroup				
R1a	2 (2%)	1 (1%)	4 (1%)	2 (1%)
R1b	146 (59%)	67 (63%)	160 (69%)	118 (66%)
R1b-DF27	117 (47%)	52 (49%)	132 (57%)	96 (54%)

**Fig. 1:**
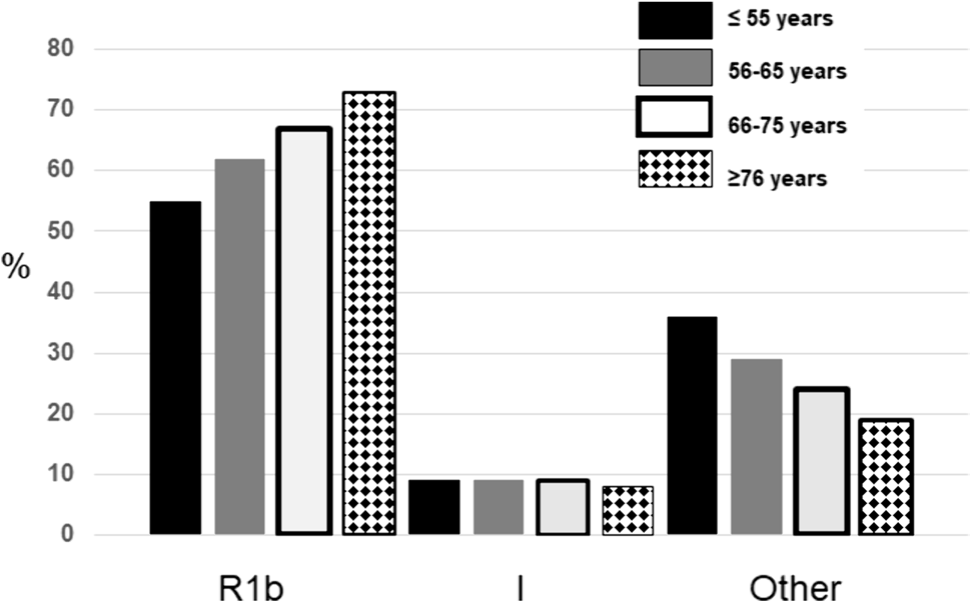
Frequencies of R1b, I and other Y-haplogroups in COVID-19 patients, four age ranges

For the non-R1b haplogroups we found higher frequencies of Y-I in the two patients compared to control groups. Y-I was found in 9% of the patients and 4% of the controls (*p* = 0.02) with OR = 2.19 (95%CI = 1.13–4.22). Thus, in our population haplogroup Y-I could represent a risk marker for severe COVID-19 in male, although the reduced frequency of this haplogroup in our population is a study limitation. We compared the R1b and I between four age groups in the patients. While Y-I frequencies did not differ between the groups the R1b frequency showed an increased value with the age, ranging from 55% in the patients ≤55 years (*N* = 118) to 73% in patients aged >76 years (*N* = 92) (Fig. 1; Suppl. Table 1).

Hypertension is a well characterized risk factor for developing severe COVID-19. We sought to investigate whether the Y-haplogroups were associated with the presence of hypertension in our patients and controls. We found higher frequencies of R1b among the hypertensives in the two patients groups (≤65 years, *p* = 0.16; >65 years, *p* = 0.04), without difference between hypertensive and normotensive controls (Table 4).This suggested that individuals with pre-existing hypertension who are Y-R1b could have an increased risk of severe COVID-19 (Suppl. Table 2). In reference to Y-I, we found no different frequencies in the two patients groups regardless of the hypertension status.

**Table 4:** Y-haplogroup frequencies in the COVID-19 patients according to the presence of hypertension

Y-haplogroup	≤65 years			
	Patients		Controls	
	Hypertensives *N* = 103	Normotensives *N* = 145	Hypertensives *N* = 30	Normotensives *N* = 77
R1b	66 (64%)	80 (55%)	17 (61%)	50 (65%)
R1a	1 (1%)	1 (1%)	1 (3%)	0
E	9 (9%)	16 (11%)	2 (6%)	8 (10%)
G	2 (2%)	3 (2%)	2 (6%)	5 (6%)
I	10 (10%)	13 (9%)	0	4 (5%)
J	8 (8%)	10 (7%)	3 (10%)	7 (9%)
Other	7 (7%)	22 (15%)	5 (13%)	3 (4%)
p-R1b vs. others	0.16		0.43	

Y-haplogroup	>65 years			
	Patients		Controls	
	Hypertensives *N* = 144	Normotensives *N* = 87	Hypertensives *N* = 60	Normotensives *N* = 118
R1b	105 (73%)	55 (63%)	40 (67%)	78 (66%)
R1a	2 (2%)	2 (3%)	0	2 (2%)
E	7 (5%)	6 (7%)	6 (10%)	12 (10%)
G	5 (3%)	2 (3%)	4 (7%)	7 (6%)
I	9 (6%)	10 (11%)	3 (5%)	4 (3%)
J	6 (4%)	3 (3%)	5 (8%)	7 (6%)
Other	10 (7%)	9 (10%)	2 (3%)	8 (6%)
p-R1b vs. others	0.04		0.92	

**Fig. 2:**
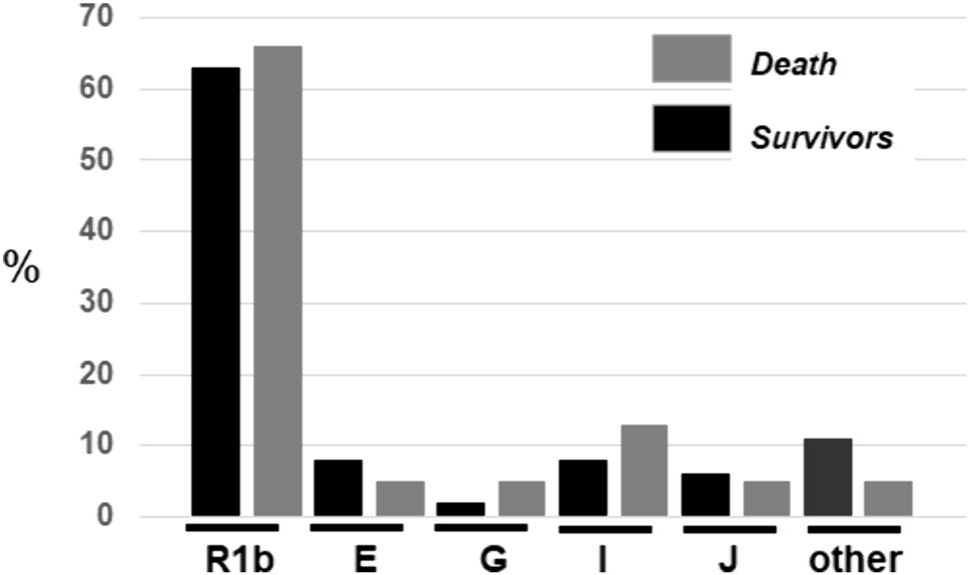
Frequencies of the Y-haplogroups in COVID-19 death and survivors

There were a total of 56 deaths (12%) among the patients, 10 in the ≤65 years (4%) and 46 in the >65 years (20%). For the whole cohort there were no difference in haplogroup frequencies between deaths and survivors (Fig. 2).

## Discussion

Chromosome-Y has been involved in the regulation of immune pathways, and this could explain in part the differences observed between male and female for several traits (Maan et al. 2017). Human chromosome-Y polymorphisms define the Y-haplogroups, which frequencies are population-specific. Several studies supported the association between some Y-haplogroups and common human diseases (Barros et al. 2020; Kuroki and Fukami 2023). Among others, haplogroup Y-I was associated with an increased risk for atherosclerosis (Charchar et al. 2012). This haplogroup was associated with significant different expression of several genes in macrophages, a cell type that plays an important role in the development of atherosclerosis (Bloomer et al. 2013). Moreover, compared to other haplogroups macrophages from Y-I men showed suppression of adaptive immunity and upregulation of pro-inflammatory response pathways (Charchar et al. 2012).

Due to the role of chromosome-Y in immunity the Y-haplogroups could be associated with differences in the extent of anti-viral responses. Rodent studies showed that the Y-chromosome modulates viral infection through RNAs that regulate the expression of several genes relevant to lymphocyte activation and recruitment (Krementsov et al. 2017). In this regard, Y-I male showed accelerated progression toward AIDS and reduced response to retroviral therapy (HAART) among HIV-infected male (Sezgin et al. 2009). Some studies showed that Y-chromosome takes part in chromatin modification mechanisms regulating the global gene expression in immune cells (Case et al. 2013). An study showed that male with Y-I had increased active chromatin sites associated with promoters and enhancers (Eales et al. 2019).

There are several mechanisms by which chromosome-Y variants could contribute to the severity of COVID-19. Male represent approximately 75% of the cases with critical COVID-19 in our population (Coto et al. 2021). The extent of the immune activity could be different between the Y-haplogroups and this would affect the response to SARS-CoV-2 among male. In the other hand, some chromosome Y variants could increase the risk for developing cardiovascular diseases that are associated with a higher risk of suffering severe COVID-19 (Bloomer et al. 2014). We found a significantly decreased frequency of R1b among the younger patients. The Y-haplogroup frequencies in our controls were similar to the reported by others, with R1b as the most common among Asturias male, and we did not find significant differences between controls aged ≤65 and >65 years. Thus, it was unlikely that the differences between the age groups of the patients could be explained by an age-effect in the general population. We found a higher frequency of R1b among patients with pre-existing hypertension. However, this haplogroup was not associated with hypertension among the controls (Table 4). This suggested that the common R1b haplogroup could be a risk factor for severe COVID-19 among hypertensives, instead of increasing the risk of hypertension in the general population. Hypertension was more frequent among the elderly patients and this could explain the higher frequency of R1b among these compared to the younger patients.

To our knowledge there are no studies supporting a significant functional effect of this haplogroup that could explain the association with COVID-19. However, some authors suggested that the rate of SARS-CoV-2 infection and mortality by COVID-19 was positively correlated with R1b, since populations with the highest R1b showed the highest rates of severe disease (Delanghe et al. 2020; Ibrahim and Salih 2021). Our study supports this hypothesis, but was based a single population and would require the genotyping of patients and controls from other European regions.

The main limitation of our study was the sample size that could reduce the power of the statistical analysis. This could be relevant for haplogroups with a low population frequency, that would require larger cohorts to confirm a genetic association. In particular, haplogroup I has a frequency of 5% in our population, much lower than that described in other populations where a positive association with cardiovascular traits was observed (Charchar et al. 2012). Our work requires validation in other populations as well as studies to determine the functional link between chromosome Y variants and COVID-19.

In conclusion, we report the chromosome Y haplogroup frequencies in controls and severe COVID-19 patients from the region of Asturias. Haplogroup Y-I could contribute to the risk of developing severe COVID-19, while haplogroup R1b was associated with an increased risk for severe disease among individuals with pre-existing hypertension. This work suggested that chromosome Y variants might serve as biological markers for the risk of developing severe COVID-19 in male.

### Supplementary Information

Below is the link to the electronic supplementary material.

Supplementary file1 (DOCX 212 KB)

## Data Availability

The data that support the findings of this study are available from the corresponding author upon reasonable request. An Excel file with the raw data would be available for meta-analysis research.
