# Supplementary material for: Chromosome-Y haplogroups in Asturias (Northern Spain) and their association with severe COVID-19
Source: Mol Genet Genomics. 2024 May 4;299(1):49. doi: 10.1007/s00438-024-02143-4 (PMC11069473; doi:10.1007/s00438-024-02143-4)
Supplement: Supplementary file 1 — Supplementary file1 (DOCX 212 KB) [file 438_2024_2143_MOESM1_ESM.docx]

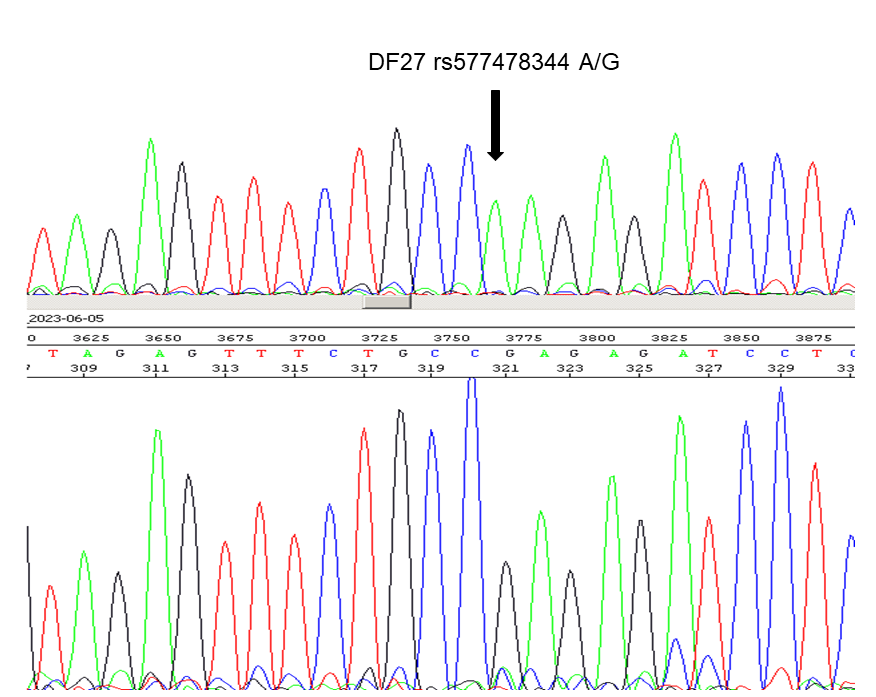


**Suppl figure 1.** Sanger sequence of PCR fragments showing the two DF27 alleles. DNA from R1b patients was PCR amplified with primers 5’TGTTAAAGTCCTGCGCTATTATGGTGT and 5’AAATATAGACGAATGCATAACTAGAATAACC (fragment size=1,312 bp). PCR fragments were sequenced with an internal primer, 5´GACAAAATCTCTCAGCATTTGC using BigDye chemistry and a capillary sequencer.


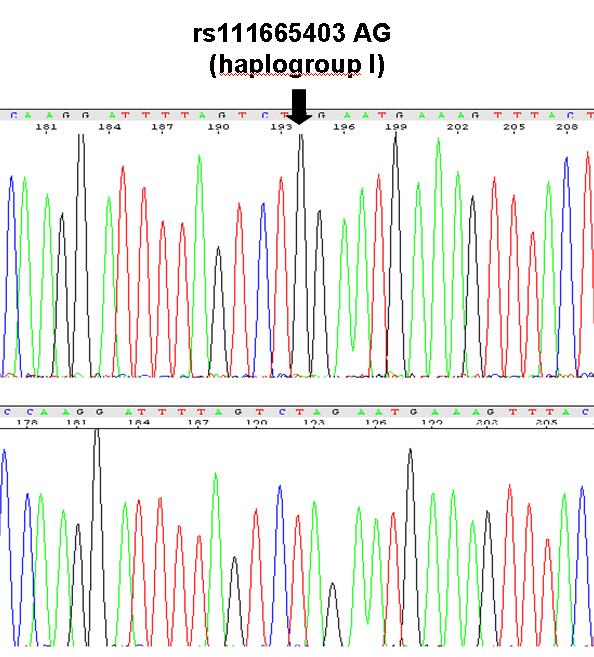


**Suppl. Figure 2.** Sanger sequence of two fragemnets showing SNP rs111665403 A/G, a surrogate marker for haplogroup I I in complete linkage disequilibrium with rs2032597. The DNA was PCR amplified with primers:

Forward CCTGGCAGGAAGTCCCTCCAAGT

Reverse GACCCCCTTGCAGGATCAGGTATCT.

**Suppl. Table 1.** Frequency of the Y haplogroups in four age-range of COVID-19 patients.

|  | **≤55 years** | **56-65 years** | **66-75 years** | **≥76 years** |
| --- | --- | --- | --- | --- |
| **R1B** | **65 (55%)** | **81 (62%)** | **93 (67%)** | **67 (73%)** |
| **R1A** | **1** | **1** | **4** | **0** |
| **E** | **13** | **12** | **4** | **9** |
| **G** | **1** | **4** | **5** | **2** |
| **I** | **11** | **12** | **12** | **7** |
| **J** | **10** | **8** | **7** | **2** |
| **OTHER** | **16** | **13** | **14** | **5** |
|  | **117** | **131** | **139** | **92** |

**Suppl. table 2.** R1b frequency in hypertensives and normotensives of the four age groups of patients.

|  | **HYPERTENSIVES** | | **NORMOTENSIVES** | |
| --- | --- | --- | --- | --- |
| **AGE** | **R1B** | **TOTAL** | **R1B** | **TOTAL** |
| **≤55,N=117** | **24 (63%)** | **38 (32%)** | **42 (53%)** | **79** |
| **55-65, N=131** | **44 (61%** | **66 (50%)** | **39 (60%)** | **65** |
| **66-75, N=139** | **63 (72%)** | **87 (63%)** | **34 (65%)** | **52** |
| **≥76, N=92** | **44 (77%)** | **57 (62%)** | **23 (66%)** | **35** |
|  |  |  |  |  |
